# Hypofractionated radiotherapy followed by rhGM-CSF enhances immunogenic cell death in a murine model of pancreatic cancer

**DOI:** 10.1590/acb407625

**Published:** 2025-10-17

**Authors:** Junli Tang, Fei Chen, Hui Xia, Chao Wang, Rui Tang, Shu Luo

**Affiliations:** 1 Suining First People’s Hospital – Department of Oncology – Suining – Sichuan – China.; 2 Southwest Medical University – School of Clinical Medicine – Luzhou – Sichuan – China.

**Keywords:** Pancreatic Neoplasms, Radiotherapy, Granulocyte-Macrophage Colony-Stimulating Factor, Immunity, Immunogenic Cell Death

## Abstract

**Purpose:**

To investigate the optimal therapeutic sequence of rhGM-CSF combined with hypofractionated radiotherapy (HFRT) in treating mouse pancreatic cancer (PC) and explore the mechanisms.

**Methods:**

A PC-bearing model was established. The antitumor effects were observed under rhGM-CSF, HFRT, rhGM-CSF + HFRT, rhGM-CSF&HFRT, and HFRT + rhGM-CSF treatments. Tumor histopathological changes were examined using hematoxylin and eosin (H&E) staining. FCM was employed to detect calreticulin (CRT), mDCs, CD4+, and CD8+ T cells. Enzyme-linked immunosorbent assay (ELISA) was used to measure HMGB1, adenosine triphosphate (ATP), interleukin- (IL)-2, IL-4, IL-8, IL-10, IL-12, IFN-γ, tumor necrosis factor (TNF)-α, and iNOS levels. IF staining was performed to detect CD31 and α-smooth muscle actin, and immunohistochemistry was used to detect vascular endothelial growth factor (VEGF), soluble VEGF receptor-1 (sVEGFR-1), hypoxia-inducible factor- (HIF)-1α, and HIF-2α expression.

**Results:**

HFRT + rhGM-CSF inhibited tumor growth, promoted tumor necrosis, and increased inflammatory cell infiltration. This regimen also significantly enhanced immunogenic cell death by inducing CRT exposure and the release of HMGB1 and ATP. Furthermore, HFRT + rhGM-CSF markedly increased proportions of mDCs, CD4+ T cells, and CD8+ T cells, and upregulated expressions of IL-2, IL-8, IL-12, IFN-γ, TNF-α, and iNOS, but not IL-4 and IL-10. Additionally, rhGM-CSF synergized with HFRT to promote the normalization of blood vessels in the PC.

**Conclusion:**

HFRT followed by rhGM-CSF had the best efficacy in PC, and the molecular mechanism may be related to immunogenic cell death.

## Introduction

Pancreatic cancer (PC) is a highly malignant solid tumor of the digestive system, with pancreatic ductal adenocarcinoma (PAAD) accounting for more than 90% of cases^1^. The risk of developing PC is positively correlated with advancing age, occurring predominantly in individuals over 50 years old^2^. The extremely low detectability of PAAD contributes to a high rate of clinical underdiagnosis. Successful diagnosis at a stage amenable to surgical resection occurs in less than 20% of cases^3^. This delay in effective intervention further restricts therapeutic options for PC.

Radiotherapy plays a critical role in the comprehensive treatment of PC. Hypofractionated radiotherapy (HFRT) is a technique characterized by high-radiation doses but fewer overall treatment sessions. Compared to conventional standard fractionated radiotherapy, HFRT offers advantages such as shorter treatment duration, higher local tumor control rates, and reduced pain^4^^,^ 5. As a result, HFRT is gaining increasing attention in PC treatment, and improving its therapeutic efficacy can significantly benefit the quality of life of PC patients.

Recombinant human granulocyte-macrophage colony-stimulating factor (rhGM-CSF) is a protein produced through genetic recombination technology. It plays a crucial role in the immune system by promoting the generation and differentiation of hematopoietic stem cells in the bone marrow into various types of white blood cells, particularly granulocytes and macrophages^6^^,^ 7. Studies have shown that rhGM-CSF can bind to specific cell surface receptors, activating downstream signaling pathways that promote cell proliferation, differentiation, and survival^8^. A phase-II randomized adjuvant study demonstrated that rhGM-CSF significantly extended the distant metastasis-free survival in patients with stages IIB, IIC, and III cutaneous melanoma^9^. Based on these findings, we hypothesized that rhGM-CSF could be an adjunctive therapy in PC treatment. However, the optimal sequencing of rhGM-CSF with HFRT is still unclear, and its molecular mechanisms require further investigation.

Radiation therapy plays a crucial role in the treatment of PC. HFRT has been shown to stimulate the immune system. Studies report that HFRT increases CD8+ T cell and IFN-γ levels while reducing the levels of suppressive Treg cells in patients with non-small cell lung cancer^10^. Enhancing and improving immune cell activity within tumor tissues is therefore a critical factor for the success of tumor immunotherapy. In recent years, rhGM-CSF has been identified as a potential adjuvant for cancer treatment due to its immunomodulatory properties. A study involving 41 patients with metastatic solid tumors observed that 11 patients experienced abscopal effects following combined treatment with rhGM-CSF and HFRT^11^. This finding suggests combining rhGM-CSF and HFRT could represent a promising anti-tumor strategy. However, this combined approach’s optimal treatment sequence and underlying molecular mechanisms remain unclear.

This study aimed to investigate the therapeutic effects and potential molecular mechanisms of different treatment regimens: rhGM-CSF and HFRT separately, rhGM-CSF + HFRT, rhGM-CSF& HFRT, and HFRT + rhGM-CSF on a PC mouse model.

## Methods

### Pancreatic cancer mice model and treatment

Panc02 (murine pancreatic cancer cells) were provided by the Department of Oncology at the Affiliated Hospital of Southwest Medical University, free of mycoplasma contamination. Panc02 cells were cultured in Dulbecco’s modified eagle medium (DMEM) supplemented with 10% fetal bovine serum until reaching the logarithmic growth phase at 37°C in 5% CO_2_. In this study, a total of 36 specific pathogen-free male C57BL/6 mice (6–8 weeks old, 18–20 g) were used, which were purchased from Chengdu Dashuo Laboratory Animals Ltd (SYXK(Chuan)2021-0246).

Cells were collected and resuspended in phosphate buffered solution (PBS), and 2×10^6^ cells were seeded into the subcutaneous right hind limb of mice and waited for tumors to reach 50–100 mm^3^. All mice were randomly divided into six groups:

- Model;
- rhGM-CSF;
- HFRT;
- rhGM-CSF + HFRT;
- rhGM-CSF & HFRT;
- HFRT + rhGM-CSF.

**Figure 1:**
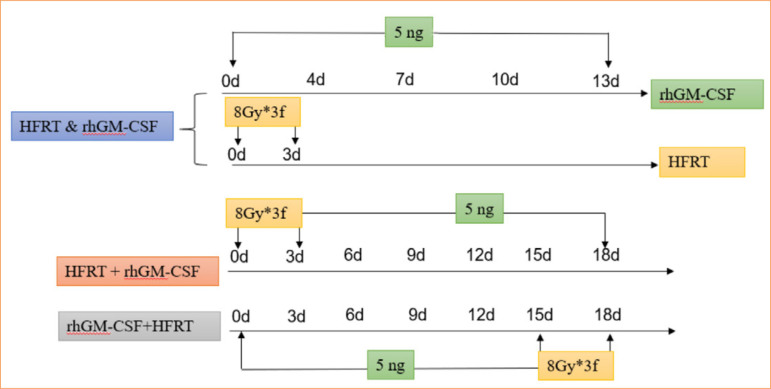
The workflow of treatment in pancreatic cancer in mice.

HFRT: hypofractionated radiotherapy; rhGM-CSF: recombinant human granulocyte-macrophage colony-stimulating factor. Source: Elaborated by the authors.

A rhGM-CSF and HFRT treatment was employed as Fig. 1. For rhGM-CSF treatment, subcutaneous injection of 5-ng recombinant protein rhGM-CSF were performed into the tumor vicinity^12^; for HFRT treatment, the body of the mouse was covered with a lead plate after anesthetizing, exposing only the tumor on its right hind limb, and treating it with a radiation dose of 8 Gy*3f^13^. The body weight was recorded every three days, and the tumor size was recorded at the end of the experiment.

Humane endpoints for mice were defined as:

- Loss of 20% body weight;
- Complete loss of appetite for 24 hours;
- Weakness preventing self-feeding and drinking;
- Persistent self-mutilation;
- Tumor volume exceeding 15 mm;
- Tumor metastasis or ulceration.

Mice were euthanized at the end of the experiments. They received an intraperitoneal dose of sodium pentobarbital (0.1 mg/100 g) for anesthesia and were then sacrificed by breaking their necks.

### Hematoxylin and eosin staining

Tumors were removed after mice were sacrificed under anesthesia, and 4% polyformaldehyde was used to fix samples. Next, samples were dried, embedded, and cut into 5-μm slices. After dewaxing, slices were stained with hematoxylin for 5 min. We rinsed the slices in running water until there was no floating color and differentiated them using the alcoholic hydrochloric acid solution for 3 s. Subsequently, slices were placed into a weak alkaline solution, followed by an eosin staining for 3 min.

### Immunohistochemistry assay

The tumor slices were prepared as previously mentioned. The tumor sections were washed three times with PBS and then incubated in 3% peroxide for 3 min, followed by incubation in 5% bovine serum albumin (BSA) at room temperature for 30 min. Primary antibodies against hypoxia-inducible factor-1α (HIF-1α) (20960-1-AP, Proteintech), hypoxia-inducible factor-2α (HIF-2α) (PA1-16510, Thermo Fisher Scientific), soluble vascular endothelial growth factor (VEGF) receptor-1 (sVEGFR-1) (36-1100, Thermo Fisher Scientific), and VEGF (19003-1-AP, Proteintech) were used to incubate with tumor sections overnight at 4°C. After three times of PBS washing, it was then incubated with the HRP-labeled goat anti-rabbit (GB22303, 1:100, Servicebio) for 30 min at room temperature. 3,3′-Diaminobenzidine (DAB) was added to colorize the sections, and a fluorescence microscope (VS200, OLYMPUS, Japan) was employed to visualize it.

### Flow cytometry

Tumor tissues were rinsed, cut into small pieces, and ground with a grinder. The cell suspension was collected and filtered through a 200-mesh cell sieve and centrifuged at 300 g for 5 min. The cell sediment was washed twice with PBS, and 100-μL PBS was used to resuspend cells in each tube. FITC anti-mouse CD4 (no. 100405, Biolegend), PE anti-mouse CD8 (no. 162303, Biolegend), PerCP/Cyanine5.5 anti-mouse CD86 (no. 105027, Biolegend), PE anti-mouse CD80 (no. 104707, Biolegend), APC anti-mouse CD11c (no. 117309, Biolegend), MHC II (no. 107605, Biolegend), and calreticulin (CRT) (no. SPC-122B-FITC, stressmarq) antibodies were used to label cells, respectively. Cells were incubated for 30 min at 4°C in the dark followed by PBS washing and centrifuging at 300 g for 5 min. We resuspended the cells in PBS, then detected and analyzed them on the flow analyzer (Cytoflex, Beckman).

### Immunofluorescence double staining

The tumor sections were washed three times with PBS and then incubated in 3% peroxide for 3 min, followed by incubation in 5% BSA at room temperature for 30 min. Primary antibodies against CD31 (no. ab182981, 1:1,000, Abcam) and α-smooth muscle actin (SMA) (no. ab5694, 1:200, Abcam) were used to incubate the tumor sections overnight at 4°C. After three times of PBS washing, it was then incubated with the FITC-labeled goat anti-rabbit (GB22303, 1:100, Servicebio) and CY3-labeled goat anti-rabbit (GB21303, 1:200, Servicebio) for 30 min at room temperature. Lastly, DAPI was used to stain sections for 10 min at room temperature. All samples were photographed with a fluorescence microscope (VS200, Olympus, Japan).

### Enzyme-linked immunosorbent assay

The tumor tissue was prepared as a homogenate mixture using a tissue grinding instrument. The samples were centrifuged at 5,000 g for 10 min, and the supernatant liquid was collected for enzyme-linked immunosorbent assay (ELISA) detection. ELISA kits including mouse IL-2 ELISA kit (ZC-37976, ZIO), mouse IL-4 ELISA kit (ZC-37986, ZIO), mouse IL-8 ELISA kit (ZC-37953, ZIO), mouse IL-10 ELISA kit (ZC-37962, ZIO), mouse IL-12 ELISA kit (ZC-37964, ZIO), mouse iNOS ELISA kit (ZC-38979, ZIO), mouse IFN-γ ELISA kit (ZC-37905, ZIO), mouse TNF-α ELISA kit (ZC-39024, ZIO), mouse HMGB1 ELISA kit (ZC-38180, ZIO) were used in this study.

### Adenosine triphosphate detection

Tumor samples were collected and ground. After centrifugation of 5,000 g for 10 min, the supernatant was collected to measure the adenosine triphosphate (ATP) levels of each group with an ATP kit (A095-1-1, Nanjing Jiancheng).

### Statistical data analysis

Data analysis was conducted with Statistical Package for the Social Sciences 27.0 and GraphPad Prism 9.0. Results are presented as mean ± standard deviation, and comparisons among three or more groups utilized one-way analysis of variance (ANOVA). Group mean comparisons employed the least significant difference method, considering *p* < 0.05 as the threshold for statistical significance.

## Results

### Efficacy of rhGM-CSF combined with hypofractionated radiotherapy in the treatment of pancreatic cancer tumors in mice

**Figure 2:**
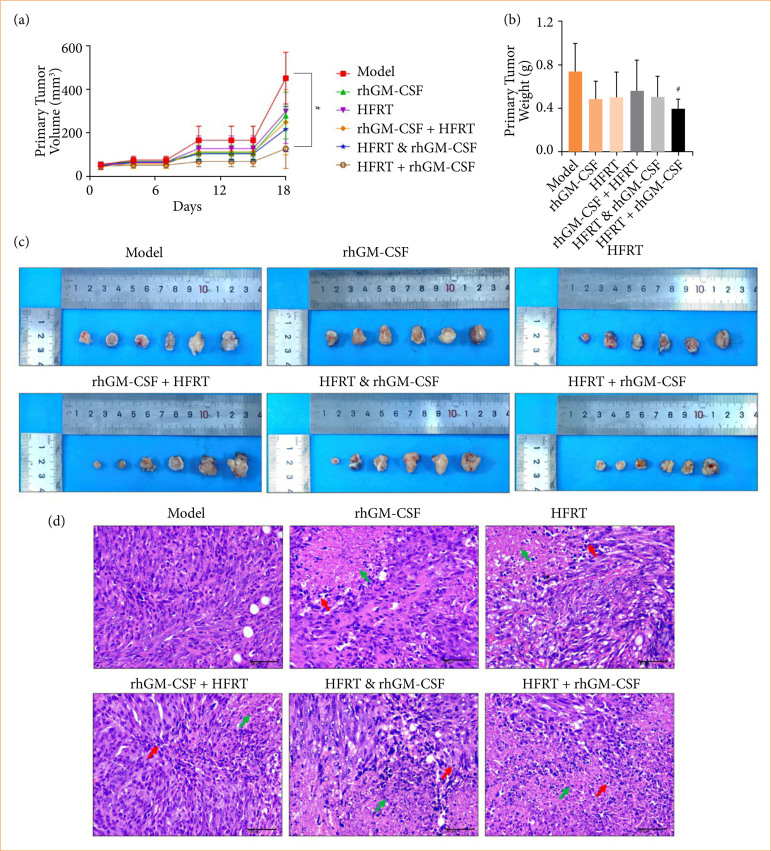
rhGM-CSF treatment followed by HFRT has the best efficacy in PC tumors than other combination orders. Tumors were treated with rhGM-CSF and HFRT, respectively, or in combination (HFRT followed by rhGM-CSF treatment, HFRT & rhGM-CSF treatment concurrently, and rhGM-CSF treatment followed by HFRT). **(a)** tumor volume (mm^3^). **(b)** tumor weight **(g)**. **(c)** primary tumor. **(d)** Hematoxylin and eosin staining.

HFRT: hypofractionated radiotherapy; rhGM-CSF: recombinant human granulocyte-macrophage colony-stimulating factor; PC: pancreatic cancer; #*p* < 0.05, when compared with the model group. Bar = 50 μm. Source: Elaborated by the authors.

During the whole treatment cyclical, the primary tumor volume was recorded (Fig. 2a). On day 18, the tumor volume of the HFRT+ rhGM-CSF group was significantly smaller than the model group. Likewise, at the end of the treatment day, the tumor weight trend was consistent with the volume one (Figs. 2b and 2c). We further visualized the pathological injury by hematoxylin and eosin staining. As Fig. 2d shows, the cells in the tumor tissue of the model group were tightly arranged and orderly, without necrosis. Compared with the model group, all treatment groups displayed different levels of PC cell necrosis and lymphocyte-based inflammatory cell infiltration. Importantly, the HFRT + rhGM-CSF group was found to cause the most serious PC cell necrosis.

### rhGM-CSF combined with hypofractionated regulates the vascular development in pancreatic cancer tumors

**Figure 3:**
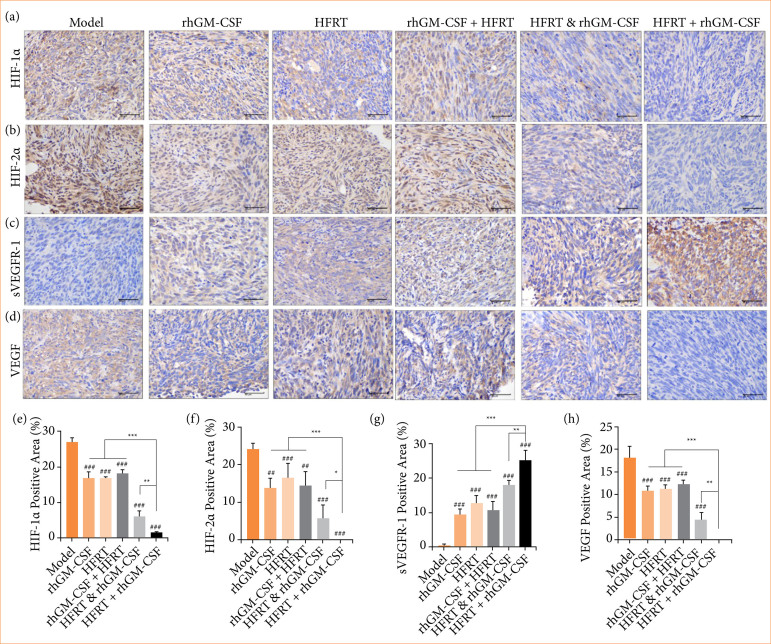
rhGM-CSF combined with HFRT inhibits the angiogenesis-related factors expression in PC tumors. Tumors were treated with rhGM-CSF and HFRT, respectively, or in combination (HFRT followed by rhGM-CSF treatment, HFRT & rhGM-CSF treatment concurrently, and rhGM-CSF treatment followed by HFRT). (**a** and **e**) HIF-1α, (**b** and **f**) HIF-2α, (**c** and **g**) sVEGFR-1, (**d** and **h**) VEGF were measured by immunohistochemistry assay.

HFRT: hypofractionated radiotherapy; rhGM-CSF: recombinant human granulocyte-macrophage colony-stimulating factor; PC: pancreatic cancer; HIF: hypoxia-inducible factor; sVEGFR-1: soluble vascular endothelial growth factor receptor-1; VEGF: vascular endothelial growth factor; ##*p* < 0.01 and ###*p* < 0.001, compared with the model group; *p < 0.05, **p < 0.01, ***p < 0.001, compared with the HFRT + rhGM-CSF group. Source: Elaborated by the authors.

Vascular normalization is an essential indicator of whether tumor suppression is occurring or not. Therefore, angiogenesis-related factors, including HIF-1α (Fig. 3a), HIF-2α (Fig. 3b), sVEGFR-1 (Fig. 3c), and VEGF (Fig. 3d), were measured by immunohistochemistry assay. The statistical results showed that HIF-1α, HIF-2α, and VEGF expressions were significantly downregulated, while sVEGFR-1 expression was upregulated in response to treatment with rhGM-CSF, HFRT, rhGM-CSF + HFRT, HFRT & rhGM-CSF, or HFRT+rhGM-CSF. Among all treatment groups, the HFRT + rhGM-CSF regimen resulted in the most pronounced downregulation of HIF-1α, HIF-2α, and VEGF, as well as the most significant upregulation of sVEGFR-1.

**Figure 4:**
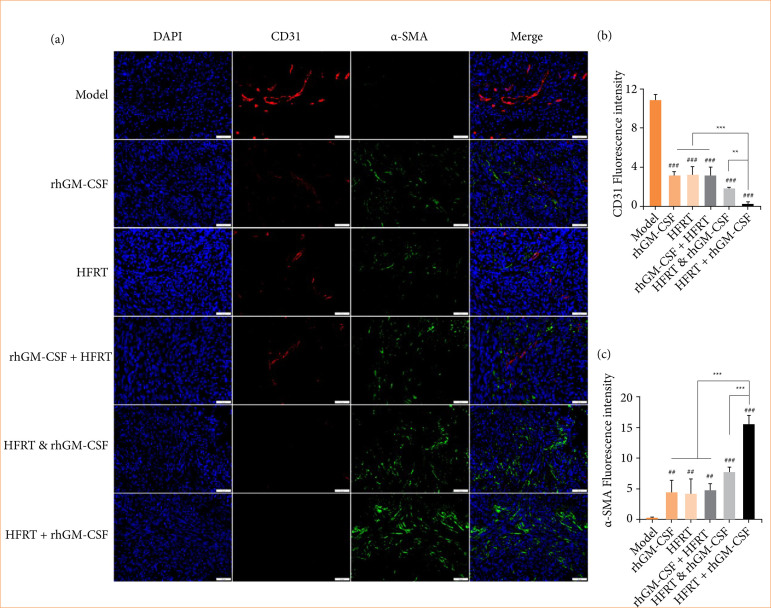
rhGM-CSF combined with HFRT decreases the CD31 and increases α-SMA expression in PC tumors. Tumors were treated with rhGM-CSF and HFRT, respectively, or in combination (HFRT followed by rhGM-CSF treatment, HFRT & rhGM-CSF treatment concurrently, and rhGM-CSF treatment followed by HFRT). **(a)** IF double staining was performed to visualize the CD31 and α-SMA expression. **(b)** The bar chart of CD31 expression. **(c)** The bar chart of α-SMA expression.

HFRT: hypofractionated radiotherapy; rhGM-CSF: recombinant human granulocyte-macrophage colony-stimulating factor; PC: pancreatic cancer; SMA: smooth muscle actin; ##*p* < 0.01 and ###*p* < 0.001, compared with the model group; **p < 0.01, ***p < 0.001, compared with the HFRT + rhGM-CSF group. Source: Elaborated by the authors.

To evaluate the vascular normalization level of the PC tumor in each group, IF double staining was performed to visualize the CD31 and α-SMA expression (Fig. 4a). As Figs. 4b and 4c show, the CD31 expression was significantly downregulated, while α-SMA expression was upregulated in response to the treatment with rhGM-CSF, HFRT, rhGM-CSF + HFRT, HFRT & rhGM-CSF, and HFRT + rhGM-CSF. Importantly, the HFRT + rhGM-CSF group revealed the greatest effect.

### rhGM-CSF combined with hypofractionated regulates the immunogenic cell death in pancreatic cancer tumors

**Figure 5:**
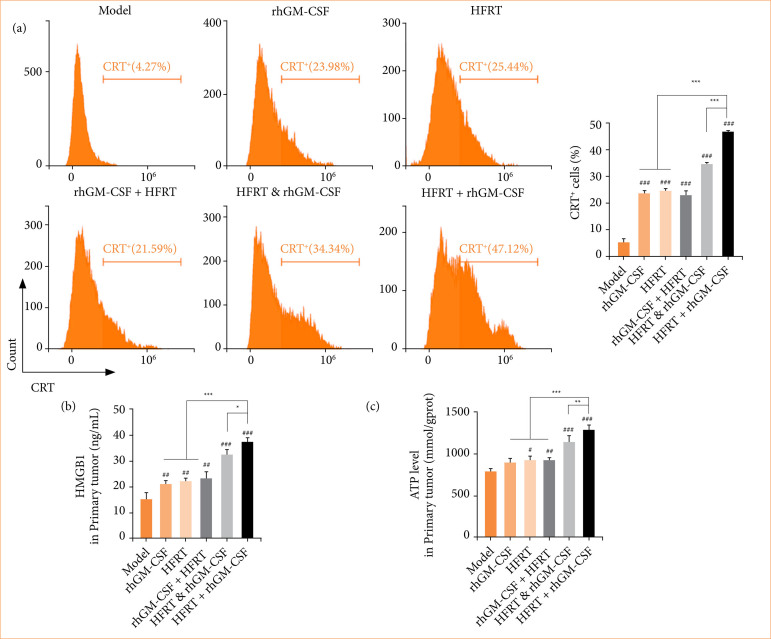
rhGM-CSF combined with HFRT regulates immunogenic cell death in PC tumors. Tumors were treated with rhGM-CSF and HFRT respectively, or in combination (HFRT followed by rhGM-CSF treatment, HFRT & rhGM-CSF treatment concurrently, and rhGM-CSF treatment followed by HFRT). **(a)** Flow cytometry was conducted to quantify the CRT+ cell ratio in each group. **(b)** Enzyme-linked immunosorbent assay was performed to detect the HMGB1 level. **(c)** The ATP kit was used to quantify the ATP level.

HFRT: hypofractionated radiotherapy; rhGM-CSF: recombinant human granulocyte-macrophage colony-stimulating factor; PC: pancreatic cancer; ATP: adenosine triphosphate; #*p* < 0.05, ##*p* < 0.01 and ###*p* < 0.001, compared with the model group; *p < 0.05, **p < 0.01, ***p < 0.001, compared with the HFRT + rhGM-CSF group. Source: Elaborated by the authors.

Flow cytometry was used to determine the CRT+ cell ratios in tumors (Fig. 5a). Results revealed that CRT+ cell ratios were upregulated to varying degrees in all treatment groups in comparison to the model group. ELISA was performed to detect the high-mobility group box 1 (HMGB1) level, and an ATP kit was used to quantify the ATP level. Figures 5b and 5c show that HMGB1 and ATP levels were both increased by treatment of rhGM-CSF, HFRT, rhGM-CSF + HFRT, HFRT & rhGM-CSF, or HFRT + rhGM-CSF. Interestingly, the HFRT + rhGM-CSF group induced the highest CRT+ cell ratio, HMGB1, and ATP levels.

### Effect of rhGM-CSF combined with hypofractionated radiotherapy on immunogenicity in pancreatic cancer tumors

**Figure 6:**
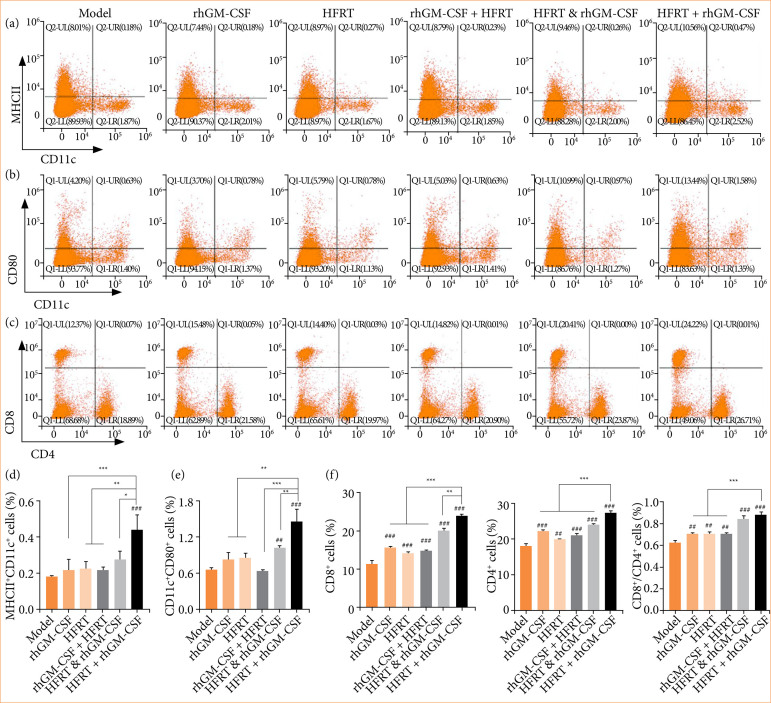
rhGM-CSF combined with HFRT increases the mDC and T cell levels. Tumors were treated with rhGM-CSF and HFRT, respectively, or in combination (HFRT followed by rhGM-CSF treatment, HFRT & rhGM-CSF treatment concurrently, and rhGM-CSF treatment followed by HFRT). **(a)** D1 mDC, **(b)** D2 mDC, and **(c)** T cells were measured by flow cytometry. **(d)** CD11c+/MHCII+ cells. **(e)** CD11c+/CD80+ cells. **(f)** CD4+ T cells, CD8+ T cells, and CD4+/CD8+ T cells.

HFRT: hypofractionated radiotherapy; rhGM-CSF: recombinant human granulocyte-macrophage colony-stimulating factor; PC: pancreatic cancer; #*p* < 0.05, ##*p* < 0.01 and ###*p* < 0.001, compared with the model group; *p < 0.05, **p < 0.01, ***p < 0.001, compared with the HFRT + rhGM-CSF group. Source: Elaborated by the authors.

To investigate the immunogenicity changes in PC tumors under various treatments, a flow cytometry assay was performed. CD11c+/MHCII++ identified D1 subtype mDC cells (Fig. 6a), CD11c+/CD80+ identified D2 subtype mDC cells (Fig. 6b), and CD4+/CD8+ identified T cells (Fig. 6c). As shown in Figs. 6d, 6e, and 6f, the levels of D1 and D2 mDC cells, CD4+ T cells, CD8+ T cells, and CD4+/CD8+ T cells were increased across all treatment groups, including rhGM-CSF, HFRT, rhGM-CSF & HFRT, and rhGM-CSF + HFRT. The most significant upregulation was observed in the HFRT + rhGM-CSF treatment group.

**Figure 7:**
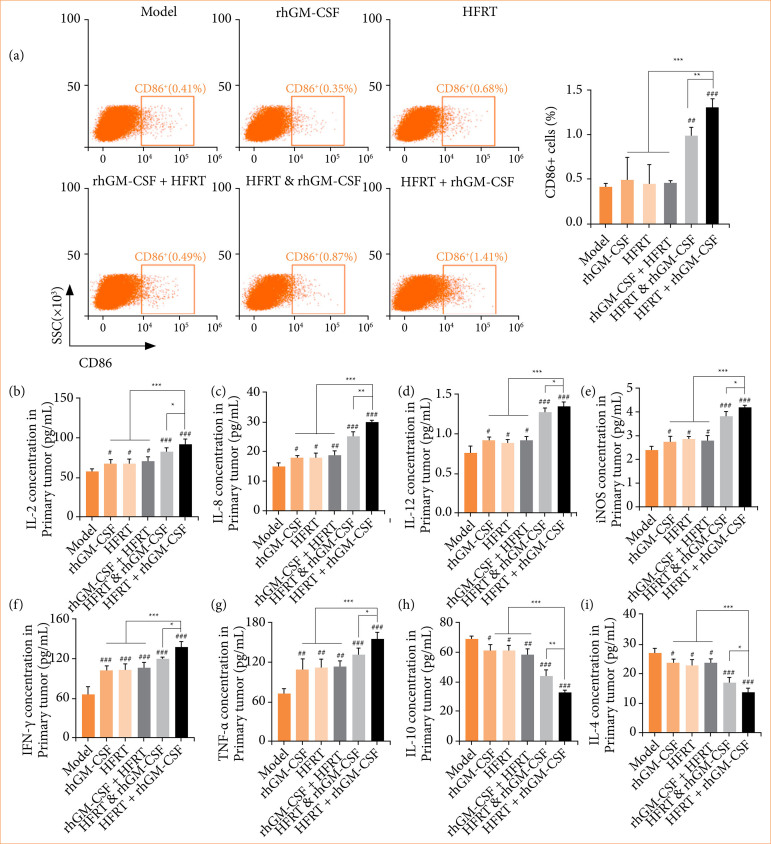
rhGM-CSF combined with HFRT regulates the M1 macrophages and inflammation-related cytokines expression. Tumors were treated with rhGM-CSF and HFRT, respectively, or in combination (HFRT followed by rhGM-CSF treatment, HFRT & rhGM-CSF treatment concurrently, and rhGM-CSF treatment followed by HFRT). **(a)** Flow cytometry was conducted to count the M1 macrophages (%). Enzyme-linked immunosorbent assays were used to measure the inflammation-related cytokine levels, including **(b)** IL-2, **(c)** IL-8, **(d)** IL-12, **(e)** iNOS, **(f)** IFN-γ, **(g)** tumor necrosis factor (TNF)-α, **(h)** IL-10, **(i)** IL-4.

HFRT: hypofractionated radiotherapy; rhGM-CSF: recombinant human granulocyte-macrophage colony-stimulating factor; PC: pancreatic cancer; IL: interleukin; iNOS; IFN: interferon; TNF: tumor necrosis factor; #p < 0.05, ##p < 0.01 and ###p < 0.001, compared with the model group; *p < 0.05, **p < 0.01, ***p < 0.001, compared with the HFRT + rhGM-CSF group. Source: Elaborated by the authors.

To further explore the immunogenicity changes, the M1 macrophages and inflammation-related cytokines in PC tumors were quantified. Figure 7a shows that the rhGM-CSF, HFRT, and rhGM-CSF + HFRT groups had no significant effect on the number of M1 macrophages. However, HFRT & rhGM-CSF and HFRT + rhGM-CSF treatments increased the number of M1 macrophages. Furthermore, inflammation-related cytokines, including interleukin (IL)-2 (Fig. 7b), IL-8 (Fig. 7c), IL-12 (Fig. 7d), iNOS (Fig. 7e), interferon (IFN)-γ (Fig. 7f), and tumor necrosis factor- (TNF)-α (Fig. 7g) were upregulated by treatment of rhGM-CSF, HFRT, rhGM-CSF + HFRT, HFRT & rhGM-CSF, or HFRT + rhGM-CSF, but IL-4 (Fig. 7h) and IL-10 (Fig. 7i) levels were decreased. Importantly, the HFRT + rhGM-CSF group revealed the greatest effect.

## Discussion

To investigate whether rhGM-CSF can enhance the efficacy of HFRT in treating PC in mice and to determine the optimal sequence, this study evaluated various treatment regimens. These included HFRT, rhGM-CSF, rhGM-CSF + HFRT, HFRT & rhGM-CSF, and HFRT + rhGM-CSF. The results showed that HFRT + rhGM-CSF group resulted in the most pronounced tumor necrosis and inflammatory cell infiltration, as well as significant tumor shrinkage. It indicates that rhGM-CSF can effectively enhance the PC therapeutic efficacy of HFRT. Administration of 5ng rhGM-CSF after treatment with 8Gy*3f was more efficient in all parameters evaluated.

Abnormal angiogenesis is associated with various malignant tumors^14^^,^ 15. Solid tumors depend on a continuous vascular supply to deliver oxygen and nutrients essential for their growth. In the tumor microenvironment, increased production of VEGF stimulates a self-reinforcing cycle of non-productive sprouting angiogenesis^16^^,^ 17. Therefore, this study quantitatively assessed VEGF and sVEGFR-1 expression in tumors using immunogenic cell death. The results revealed that the HFRT + rhGM-CSF treatment significantly reversed the high expression of VEGF observed in the model group while increasing the low expression of sVEGFR-1 in the same group. This study demonstrated the effectiveness of HFRT + rhGM-CSF in inhibiting intratumoral angiogenesis in a PC mouse model.

Platelet endothelial cell adhesion molecule-1 (CD31), a highly glycosylated immunoglobulin-like membrane receptor, has been reported to facilitate cell migration, signal transduction, and tumor angiogenesis while inhibiting apoptosis^18^^,^ 19. Mature blood vessels contain various contractile proteins, including α-SMA, commonly used as a pericyte marker^20^. The process of vascular maturation involves the encapsulation of nascent vascular sprouts by α-SMA+ pericytes^21^.

This study further revealed that the HFRT + rhGM-CSF treatment significantly reduced CD31 expression while upregulating α-SMA. Additionally, substantial evidence supports that HIF-1α and HIF-2α are essential proteins for angiogenesis and cancer metastasis^22^^-^ 24. Immunohistochemical analysis of HIF-1α and HIF-2α expression in this study corroborated these findings. These results indicate that the HFRT + rhGM-CSF treatment promotes the normalization of tumor vasculature in PC.

Therapeutic approaches such as radiotherapy, recombinant proteins, or immune checkpoint inhibitors can induce a form of tumor-specific cell death known as ICD. The hallmark of ICD is the active or passive release of damage-associated molecular patterns (DAMPs)^25^^,^ 26, including the exposure of CRT on the surface of dying cells, the release of large amounts of ATP, and the translocation of HMGB1 from the nucleus to the cytoplasm. These DAMPs are recognized by antigen-presenting cells, which subsequently activate the immune system to elicit a tumor-specific immune response^27^. In this study, HFRT + rhGM-CSF treatment significantly upregulated CRT, ATP, and HMGB1 levels, indicating that this therapeutic regimen effectively induces ICD in PC.

Studies have reported that DAMP molecules synergistically enhance the ability of dendritic cells (DCs) to phagocytose tumor cells, thereby initiating CD8+ T cells and accelerating the presentation of tumor antigens^28^. Upon activation, CD8+ T cells recognize antigens on the surface of tumor cells and directly induce apoptosis through the perforin and granzyme release^29^^,^ 30. Additionally, CD8+ T cells can inhibit tumor cell growth by secreting cytokines such as IFN-γ and TNF-α^31^. CD4+ T cells, a helper T cell, also play a pivotal role in tumor immunity. By secreting cytokines like IL-2 and IFN-γ, CD4+ T cells activate dendritic cells and macrophages, enhancing their ability to present tumor antigens and promoting the activation of CD8+ T cells^32^^,^ 33.

It was found that dendritic cells, CD4+ T cells, CD8+ T cells, and M1 macrophages were all significantly upregulated following HFRT + rhGM-CSF treatment. These findings demonstrated that rhGM-CSF enhances the ICD effect induced by HFRT and promotes pro-inflammatory responses within the tumor. ELISA results further confirmed this conclusion, showing that HFRT combined with rhGM-CSF significantly downregulated the expression of immunosuppressive factors (IL-4 and IL-10), while markedly upregulating the expression of IL-2, IL-8, IL-12, IFN-γ, TNF-α, and iNOS.

## Conclusion

The combination of HFRT and rhGM-CSF benefits ICD in PC tumors, and the HFRT + rhGM-CSF is the optimal treatment sequence.

## Data Availability

The data supporting the findings of this study are available from the first author upon reasonable request.
